# Colon-Restricted Phosphatase and Tensin Homolog Deleted From Chromosome 10 Haploinsufficiency Models Phosphoinositide 3-Kinase Pathway-Driven Invasion in Colorectal Cancer

**DOI:** 10.1016/j.jcmgh.2026.101773

**Published:** 2026-03-25

**Authors:** Haruki Sada, Hiroaki Niitsu, Yuji Urabe, Hikaru Nakahara, Masatoshi Kochi, Naoya Sakamoto, Yusuke Sotomaru, Hirotaka Tashiro, Shiro Oka, Hideki Ohdan, Eric R. Fearon, Takao Hinoi

**Affiliations:** 1 Department of Surgery, NHO Kure Medical Center and Chugoku Cancer Center, Kure, Hiroshima, Japan; 2 Department of Gastroenterological and Transplantation Surgery, Graduate School of Biomedical and Health Sciences, Hiroshima University, Higashihiroshima, Hiroshima, Japan; 3 Department of Clinical and Molecular Genetics, Hiroshima University Hospital, Hiroshima, Japan; 4 Department of Gastroenterology, Graduate School of Biomedical and Health Sciences, Hiroshima University, Higashihiroshima, Hiroshima, Japan; 5 Department of Gastroenterological Surgery, NHO Higashihiroshima Medical Center, Higashihiroshima, Hiroshima, Japan; 6 Department of Pathology and Clinical Laboratories, National Cancer Center Hospital East Japan, Kashiwa, Chiba, Japan; 7 Division of Pathology, Exploratory Oncology Research and Clinical Trial Center, National Cancer Center, Kashiwa, Chiba, Japan; 8 Natural Science Center for Basic Research and Development, Hiroshima University, Higashihiroshima, Hiroshima, Japan; 9 Department of Internal Medicine, Human Genetics, and Pathology, University of Michigan, Ann Arbor, Michigan

**Keywords:** Colorectal Cancer, Haploinsufficiency, Invasion, PTEN

## Abstract

**Background & Aims:**

The multistep accumulation of driver gene mutations is associated with early-stage colorectal tumorigenesis. To observe the phenotype-genotype correlation, we developed a series of genetically engineered mouse models based on *Apc* inactivation led by *CDX2P-Cre* and found invasive adenocarcinomas developed by inducing the loss of one *Pten* copy with *Apc* inactivation in the colonic epithelium. Here, we aimed to study the mechanisms underlying *Pten* haploinsufficiency and its clinical relevance.

**Methods:**

Tumor number, volume, and histology were compared between *CDX2P-Cre;Apc*^*flox/+*^ (*CPC;Apc*) and *CDX2P-Cre;Apc*^*flox/+;*^*Pten*^*flox/+*^ (*CPC;ApcPten*) mice, and a multi-omics analysis was used to investigate the mechanism of the invasive phenotype in *CPC;ApcPten* mice. Rapamycin was administered to tumor organoids and in vivo to evaluate its dependency on the ATK serine-threonine protein kinase (AKT)- mechanistic target of rapamycin complex 1 (mTORC1) pathway. We also performed phylogenetic analysis of tumor evolution during human colorectal carcinogenesis using multiple targeted biopsies.

**Results:**

A greater number of tumors developed in *CPC;ApcPten* mice, half of which invaded the submucosal layer. Although both Apc alleles were inactivated by Cre-LoxP recombination and loss of heterozygosity in tumors, a wild-type *Pten* allele remained in the tumors of *CPC;ApcPten* mice, suggesting the haploinsufficient effect of *Pten* on tumorigenesis. Loss of one *Pten* copy decreased *Pten* expression levels and induced downstream activation of the AKT-mTORC1 signaling pathway in *CPC;ApcPten* mice. Rapamycin administration markedly inhibited tumorigenesis in *CPC;ApcPten* and *CPC;Apc* mice. In clinical biopsies, phosphoinositide 3-kinase (PI3K) genetic alteration was predominantly observed during the transition from tubular adenoma to adenocarcinoma.

**Conclusions:**

The effect of *Pten* haploinsufficiency on colonic tumor formation in mice reaffirms the significance of PI3K alterations in tumor evolution in human colorectal cancers.

## Graphical abstract

**Figure:**
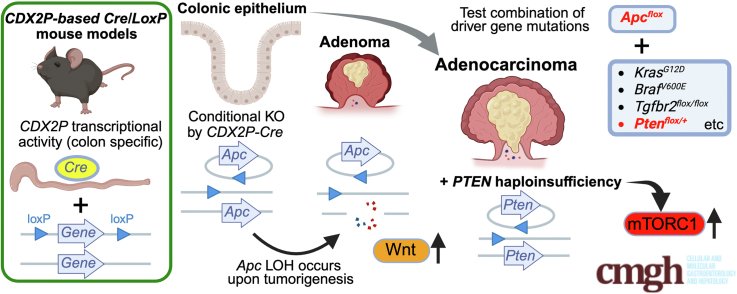

**Summary**

*CDX2P-Cre*–based genetically engineered mouse models for colonic neoplasia recapitulate diverse histological subtypes. Among them, *Pten* haploinsufficiency accelerates *Apc*-driven invasive adenocarcinoma through mechanistic target of rapamycin complex 1 activation. Consistently, human colorectal cancers exhibit phosphoinositide 3-kinase pathway activation associated with invasive progression.

**What You Need to Know**

**Background**

*CDX2P-Cre*–based colon-restricted genetically-engineered mouse models (GEMMs) provide a robust platform for dissecting genotype–phenotype relationships in colorectal tumorigenesis. However, the molecular determinants governing the early transition to invasive adenocarcinoma remain unclear.

**Impact**

*Apc* loss plus *Pten* haploinsufficiency generates a reproducible invasive phenotype in colon-restricted GEMMs. Parallel NBI-guided human biopsies demonstrate phosphoinositide 3-kinase (PI3K) pathway abnormalities linked to early invasive transformation in colorectal tumor evolution.

**Future Directions**

This trackable GEMM enables longitudinal dissection of PI3K– mechanistic target of rapamycin–driven early invasion and offers a preclinical platform to evaluate rational intervention strategies aimed at intercepting colorectal cancer progression.

Colorectal cancers (CRCs) occur as a result of multiple genetic and epigenetic events, and a number of driver gene mutations have been identified thus far, including those in *APC*, *KRAS*, and *TP53*.^1^, ^2^, ^3^ Although it has been accepted that genetic and genomic events increase linearly as adenoma transforms to adenocarcinoma, recent computational analysis considering intratumor heterogeneity based on next-generation sequencing offers much more complicated models in which nonlinear and neutral evolution are also included.^4^, ^5^, ^6^, ^7^, ^8^, ^9^ Nevertheless, clonal evolution induced by driver gene mutations still plays an important part while a normal epithelial cell transforms to a premalignant lesion and an early-stage adenocarcinoma finally develops.^6^^,^^10^^,^^11^

Although these computational analyses are useful for studying clonal evolution, it remains difficult to determine which genetic or genomic alterations directly contribute to a specific phenotype. From this point of view, genetically engineered mouse models (GEMMs) are the ultimate tools for studying genotype-phenotype correlations.^12^^,^^13^ The first GEMM of intestinal tumorigenesis was the *Apc*^*min/+*^ mouse model, in which Wnt/β-catenin signaling is activated by loss of heterozygosity of *Apc*, resulting in the formation of several intestinal adenomas, mainly in the small intestine.^14^^,^^15^ Although *Apc*^*min/+*^ mice have been invaluable for studying the initiation of intestinal tumorigenesis, they predominantly develop early adenomatous lesions in the small intestine and rarely progress to invasive or metastatic adenocarcinomas. Moreover, the tumor distribution differs from human disease, since patients with familial adenomatous polyposis (FAP), who harbor germline *APC* mutations, mainly develop colorectal rather than small-intestinal tumors.^16^ In addition, because *Apc*^*min/+*^ mice carry a germline mutation, *Apc* loss occurs systemically, leading to phenotypes in other organ systems such as hematopoietic cells.^17^ Therefore, there is a strong need for tissue-specific and multigenic GEMMs that better model human colorectal tumorigenesis.

In recent years, increasingly sophisticated GEMMs have been developed using intestine-specific Cre drivers, such as *Villin-CreERT2*, to introduce combinations of CRC–associated mutations, including *Apc*, *Kras*, *Trp53*, *Tgfbr2*, *Smad4, and Pten*.^18^, ^19^, ^20^, ^21^ These studies have successfully modeled invasive and even metastatic intestinal adenocarcinomas, providing valuable insight into how multiple driver mutations cooperate during tumor progression. Alongside these models, conditional approaches employing Adeno-Cre–mediated recombination in the distal colon^22^ and azoxymethane (AOM)-assisted tumorigenesis protocols^23^^,^^24^ have further contributed to reproducing invasive and advanced intestinal carcinomas. In addition, an inducible colon-specific *Car1-CreERT2* model has been reported, in which recombination occurs predominantly in the cecum and proximal colon,^25^ enabling spatially restricted analysis of tumorigenesis in that region. Stem cell-specific *Cre* drivers such as *Lgr5-EGFP-IRES-CreERT2* and *Lrig1-CreERT2* have demonstrated that *Apc* deletion in their respective stem/progenitor compartments initiates colorectal tumorigenesis, highlighting cell-of-origin diversity in early CRC development.^26^^,^^27^ Collectively, these diverse models have illuminated the cooperative and hierarchical roles of multiple oncogenic alterations and cellular origins during intestinal tumor progression.

In parallel with these efforts, we have generated GEMMs in which *Apc* is inactivated specifically in colonic epithelial cells via *CDX2P-Cre*, namely *CPC;Apc* mice.^16^ Compared with the aforementioned models,^18^, ^19^, ^20^, ^21^, ^22^, ^23^, ^24^, ^25^, ^26^, ^27^ including *Villin-CreERT2*-based, stem cell-specific *Cre*-based, and AOM-assisted GEMMs, our *CDX2P-Cre* platform provides a purely genetic system enabling recombination throughout the large intestine. Using this system, we have tested several combinations of driver gene mutations.^28^, ^29^, ^30^, ^31^, ^32^, ^33^ In this *CDX2P-Cre*-based GEMM series, we found that combined heterozygous loss of *Apc* and *Pten*—without additional mutations in *Kras* or *Trp53*—was sufficient to reproducibly induce multiple invasive adenocarcinomas within 3 months of age. This simple yet robust model highlights the tumor-promoting effect of *PTEN* haploinsufficiency in vivo and provides a genetic framework for studying phosphoinositide 3-kinase (PI3K)-mechanistic target of rapamycin (mTOR)-driven tumor invasion in CRC. Given that PI3K pathway alterations have also been implicated in the early invasive transition of human colorectal neoplasia, this model offers an experimentally tractable platform to explore such clinically relevant mechanisms.

Here, based on comprehensive genotype-phenotype correlation analyses across our *CDX2P-Cre*–driven GEMM series, we define a simple colon-restricted framework that faithfully recapitulates PI3K pathway-related invasion observed in human CRC. By integrating this genetic platform with targeted endoscopic biopsies guided by advanced narrow-band imaging (NBI) and subsequent DNA sequencing, we revealed that PI3K pathway alterations are closely associated with the onset of submucosal invasion, thereby reinforcing the biological continuum between our colon-restricted GEMM and human colorectal carcinogenesis.

## Results

### A GEMM of Heterozygous Loss of *Pten* with *Apc* Inactivation Is a Model for Invasive Colonic Adenocarcinomas

**Figure 1:**
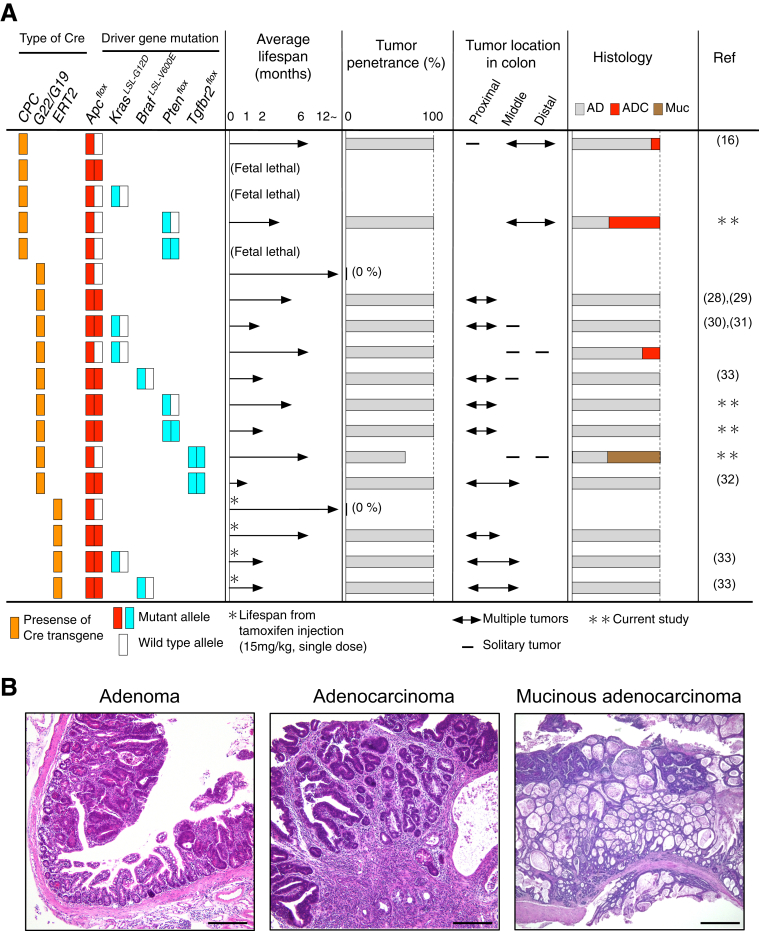
**Genetically engineered mouse models of colonic neoplasia based on Apc inactivation induced by CDX2P-Cre and derivatives.** (*A*) The genotypes of the mice, average lifespan, tumor penetrance rate, tumor location in the colon, and histology are summarized in the panel. Each combination of *Cre* driver and conditional allele (*Apc*, *Kras*, *Braf*, *Tgfbr2*, and *Pten*) is listed together with tumor incidence, anatomic distribution (proximal, middle, or distal colon), and histologic subtype (adenoma [AD], adenocarcinoma [ADC], or mucinous carcinoma [Muc]). Fetal lethality is indicated where applicable. Reference numbers correspond to previously published models, and the current study is denoted by asterisks (∗∗). (*B*) Representative histological images showing diverse tumor phenotypes observed in these models: *left*, tubular AD; *middle*, moderately differentiated ADC; *right*, mucinous adenocarcinoma (Muc). Scale bar; 500 μm. *CPC*, *CDX2P-9.5-Cre*; *G22/G19*, *CDX2P-G22-Cre* or *CDX2P-G19-Cre*; *ERT2*, *CDX2P-CreERT2*; *LSL*, *loxP-STOP-loxP*.

We previously developed several GEMMs for colonic neoplasia based on *Apc* inactivation, specifically in colonic epithelial cells, using *CDX2P-Cre*,^16^^,^^28^, ^29^, ^30^, ^31^, ^32^, ^33^ as summarized in Figure 1. We utilized the *CDX2P 9.5-NLS-Cre* mouse (*CPC*) harboring the *Cre* transgene fused to the 9.5 kb *CDX2* promoter region (*CDX2P-9.5)*, which has colonic epithelial cell-specific transcriptional activity.^16^ Colonic neoplasia efficiently develops in the *CPC;Apc*^*flox/+*^ mice (namely, *CPC;Apc*), in which most lesions exhibit adenoma. However, even in a long-term observation cohort up to 20 months of age, only 16% of mice generated invasive adenocarcinomas.^16^ To induce more advanced colonic cancers in the context of multistep carcinogenesis, we next attempted to generate *CPC;Apc*^*flox/flox*^ mice, in which *Apc* would be inactivated earlier without waiting for loss of heterozygosity, as well as *CPC;Apc*^*flox/+*^ mice with other driver gene mutations, such as *KRAS*^*LSL-G12D*^. However, neither of these mice were born because the transcriptional activity of *CDX2P-9.5* is present at the embryonic stage, and recombination of these genes during embryogenesis results in fetal lethality.^16^ To overcome this embryonic lethality, 2 *Cre* mouse lines were developed; the earlier model we developed was *CDX2P-G19/G22-Cre* mice, in which 19 or 22 guanine residues were inserted immediately after the start codon of the *Cre* transgene. Although Cre is not expressed because of a frame shift, it starts to be expressed when G19 or G22 shifts to a multiple of 3.^28^^,^^29^ This shift in G19/G22, followed by Cre expression, occurs stochastically because of DNA replication errors after the mice are born, helping them to avoid fetal lethality. The latest model—*CDX2P-CreERT2* mouse model—expresses the Cre protein fused to a mutant estrogen receptor, which cannot bind to either estradiol or estriol, but exclusively binds to tamoxifen. Cre recombinase fuse to a triple mutant form of the human estrogen receptor (Cre-ERT2) recombines with the LoxP-flanked region upon tamoxifen administration, followed by the translocation of cytoplasmic Cre-ERT2 into the nucleus.^34^^,^^35^ Tamoxifen-inducible Cre is also useful for avoiding embryonic death in GEMMs with multiple genetic alterations.^33^ Using 3 distinct *Cre* lines, we tested several combinations of driver gene mutations based on *Apc* inactivation. The tumor distribution pattern likely reflects the intrinsic transcriptional activity of the *CDX2* promoter, which is predominantly active in the proximal colon, as demonstrated by β-galactosidase staining in *Cre/LacZ* reporter mice.^16^ Accordingly, *CDX2P-G19/G22-Cre* and *CDX2P-CreERT2* models mainly develop proximal colon tumors, consistent with the physiological pattern of *CDX2P* activity. In contrast, the *CPC* model exhibits recombination throughout the colon and a relative predominance of distal tumors, which might be related to a higher frequency of *Apc* loss of heterozygosity (LOH) in the distal colon, although this possibility remains to be clarified. As for tumor histology and progression, these GEMMs mostly develop into adenomas rather than adenocarcinomas. Among previously reported GEMMs, heterozygous disruption of *Pten* in the *Apc*^*Min/+*^ background has been shown to increase tumor burden and invasiveness, predominantly in the small intestine, highlighting phosphatase and tensin homolog deleted from chromosome 10 (PTEN) dosage as a potential modifier of intestinal tumor progression.^36^ In this context of examining various combinations of driver genes, we designed new experiments to test whether the inactivation of both *Apc* and *Pten* is associated with an invasive phenotype.

### *CPC;ApcPten* Mice Develop More Invasive Colonic Neoplasia and Have a Shorter Lifespan Compared With *CPC;Apc* Mice

Initially, we expected advanced colonic neoplasia in *CPC;Apc*^*flox/+*^*;Pten*^*flox/flox*^ mice because such a tumor-suppressor gene requires the loss of both alleles. Therefore, we crossed *CDX2P-Cre;Pten*^*flox/flox*^ with *Apc*^*flox/flox*^*;Pten*^*flox/flox*^ mice to generate *CDX2P-Cre;Apc*^*flox/+*^*;Pten*^*flox/flox*^ mice. However, we did not obtain any pups, suggesting embryonic lethality in *CDX2P-Cre;Apc*^*flox/+*^*;Pten*^*flox/flox*^ mice (Figure 1*A*).

**Figure 2:**
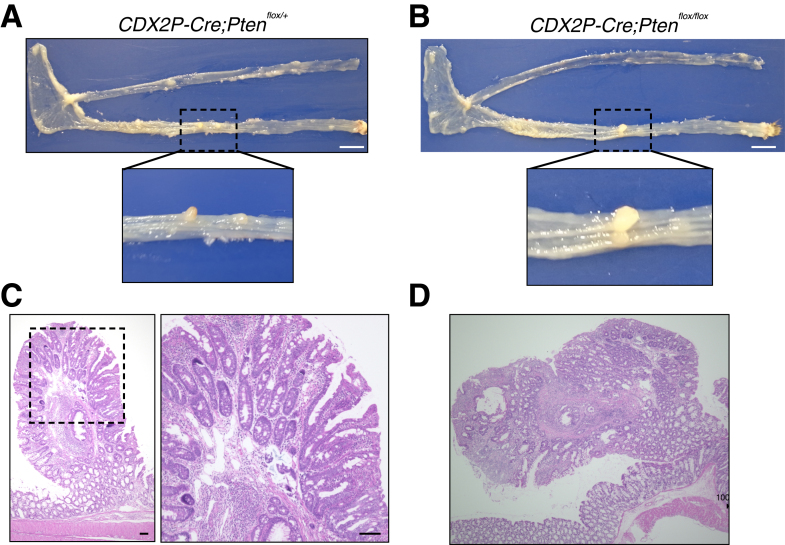
**Non-neoplastic polypoid lesions in *CDX2P-Cre;Pten*^*flox/+*^ and C*DX2P-Cre;Pten*^*flox/flox*^ mice.** (*A* and *B*) Representative gross appearance of the colon and ileum from a 25-week-old *CDX2P-Cre;Pten*^*flox/+*^ male mouse (*A*) and a 34-week-old *CDX2P-Cre;Pten*^*flox/flox*^ male mouse (*B*), showing polypoid lesions. The magnified image corresponds to the *dashed rectangle* in the gross view. Scale bar: 10 mm. (*C* and *D*) Representative histological micrographs of a non-neoplastic lesion in the same *CDX2P-Cre;Pten*^*flox/+*^ and *CDX2P-Cre;Pten*^*flox/flox*^ mice (*C* and *D*, respectively). The magnified image corresponds to the dashed rectangle in the lower-magnification panel. Scale bar: 100 μm.

We also examined the effect of colon-restricted *Pten* loss alone. *CDX2P-Cre;Pten*^*flox/+*^ mice (8 of 11; 17–44 weeks of age) and *CDX2P-Cre;Pten*^*flox/flox*^ mice (9 of 12; 8–48 weeks of age) developed a few polypoid lesions per mouse (Figure 2*A* and *B*). Histological examination revealed non-neoplastic polypoid proliferations that did not resemble either tubular adenoma or serrated lesions (Figure 2*C* and *D*). Consistent with a previous report that *Villin-Cre*–driven intestinal *Pten* deletion triggers epithelial hyperplasia without neoplastic transformation in mice up to 10 to 14 weeks of age,^37^ these findings indicate that *Pten* loss alone is not sufficient to cause tumorigenic changes in the intestinal epithelium.

**Figure 3:**
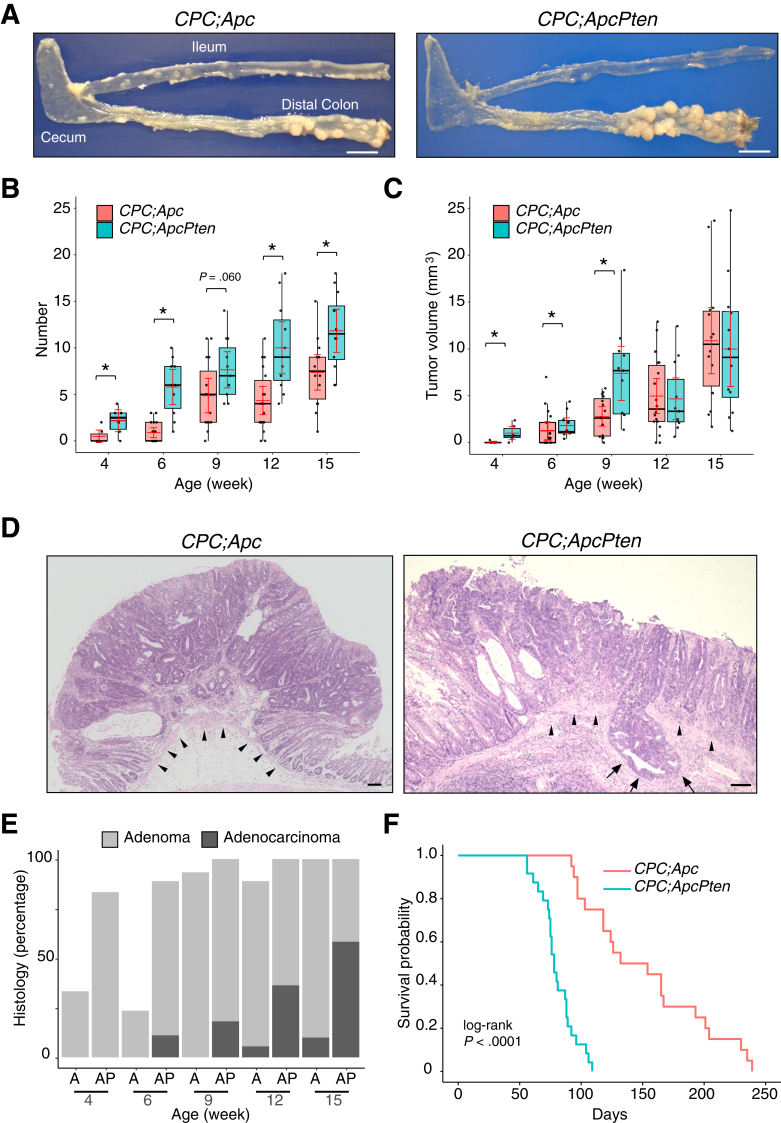
***CPC;ApcPten* mice develop more invasive colonic neoplasia and have a shorter lifespan compared with *CPC;Apc* mice.** (*A–C*) Comparison of colonic and intestinal tumor formation in *CDX2P-Cre;Apc*^*flox/+*^ (*CPC;Apc*) and *CDX2P-Cre;Apc*^*flox/+*^*;Pten*^*flox/+*^ (*CPC;ApcPten*) mice. (*A*) Gross pictures of the colon, cecum, and distal ileum at the age of 15 weeks. Scale bar: 10 mm. (*B* and *C*) Quantification of tumor number (*B*) and tumor volume (*C*). Data are presented as boxplots with individual data points, and *red lines* indicate means with 95% CIs. (*D*) Representative micrographs of H&E staining. *Arrowheads* indicate lamina muscularis mucosae. Of note, invasion beyond the lamina muscularis mucosae indicated by *arrows* was observed more frequently in *CPC;ApcPten mice*. Scale bar: 100 μm. (*E*) Percentage of mice with adenoma and adenocarcinoma among the *CPC;Apc* and *CPC;ApcPten* mice at the ages of 4, 6, 9, 12, and 15 weeks. A: *CPC;Apc*. AP: *CPC;ApcPten*. Values in (*B–E*) are derived from the same cohort. n = 6, 17, 15, 18, and 14 for *CPC;Apc* mice and n = 6, 10, 11, 11, and 12 for *CPC;ApcPten* mice at 4, 6, 9, 12, and 15 weeks, respectively. Asterisks indicate *P* < .05. (*F*) Comparison of survival in *CPC;Apc* and *CPC;ApcPten* mice (n = 20 and 24, respectively, in an independent cohort).

We next tested whether inducing the loss of one copy of *Pten* affects colonic tumor formation in the *CPC;Apc* mouse model. *CDX2P-Cre* mice were crossed with *Apc*^*flox/flox*^*;Pten*^*flox/+*^ mice to generate *CPC;Apc* and *CPC;Apc*^*flox/+*^*;Pten*^*flox/+*^ (*CPC;ApcPten*) mice from the same litters. In the time-course analysis, *CPC;ApcPten* mice developed a greater number of colonic tumors than *CPC;Apc* mice (Figure 3*A* and *B*), and exhibited larger tumor volumes at 4, 6, and 9 weeks (Figure 3*C*). These results indicate that *CPC;ApcPten* genotype enhances both tumor multiplicity and early tumor growth compared with *CPC;Apc*. More strikingly, more than one-half of the tumors in *CPC;ApcPten* mice at the age of 15 weeks showed submucosal invasion in histological analysis, in contrast to 10% of tumors with submucosal invasion in *CPC;Apc* mice at the same age (Figure 3*D* and *E*). *CPC;ApcPten* mice also had shorter lifespans than *CPC;Apc* mice (Figure 3*F*).

**Figure 4:**
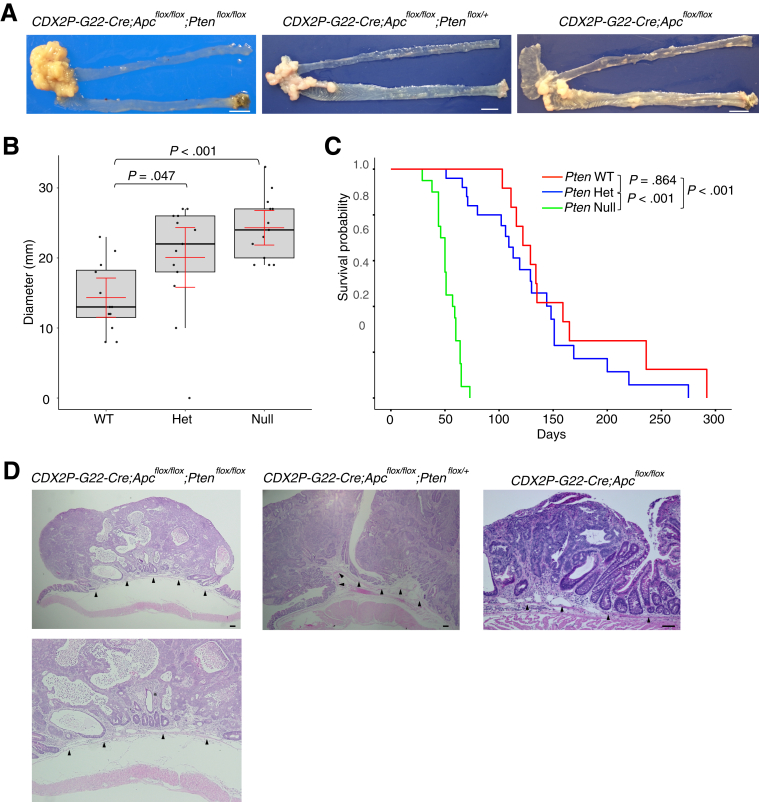
**Comparison among *CDX2P-G22-Cre;Apc*^*flox/flox*^, *CDX2P-G22-Cre;Apc*^*flox/flox*^*;Pten*^*flox/+*^, and *CDX2P-G22-Cre;Apc*^*flox/flox*^*;Pten*^*flox/flox*^ mice.** (*A*) Gross pictures of colonic and intestinal tumor formation at the age of 10 weeks. Scale bar: 10 mm. (*B*) Tumor diameter. Data are presented as boxplots with individual data points, and *red lines* indicate means with 95% CIs. n = 12, 13, and 13 for *CDX2P-G22-Cre;Apc*^*flox/flox*^ (wild-type [WT]), *CDX2P-G22-Cre;Apc*^*flox/flox*^*;Pten*^*flox/+*^ (Het), and *CDX2P-G22-Cre;Apc*^*flox/flox*^*;Pten*^*flox/flox*^ (Null) mice, respectively. (*C*) Kaplan-Meier survival analysis. n = 12, 25, and 20 for WT, Het, and Null mice, respectively. (*D*) Representative micrographs of H&E staining. For *Pten-*null mice, a higher magnification of the leading edge of the tumor is shown. There was no tumor invasion beyond the muscularis mucosae, as indicated by the *arrowheads*. Scale bar: 100 μm.

We also examined tumor-suppressor function of *Pten* in the mouse colon using another model. To measure the impact of the loss of both copies of *Pten* as well as the loss of one copy during colonic tumorigenesis, we utilized *CDX2P-G22-Cre* to avoid fetal lethality, which we had experienced in developing *CPC;Apc*^*flox/+*^*;Pten*^*flox/flox*^. As expected, *CDX2P-G22-Cre;Apc*^*flox/flox*^*;Pten*^*flox/flox*^ mice were born and developed markedly larger tumors in the proximal colon and cecum and had a shorter lifespan than *CDX2P-G22-Cre;Apc*^*flox/flox*^ mice. In addition, even the loss of one copy of *Pten* in *CDX2P-G22-Cre;Apc*^*flox/flox*^*;Pten*^*flox/+*^ mice had a significant impact on increasing the tumor size, although the lifespan of these mice was comparable to that of *CDX2P-G22-Cre;Apc*^*flox/flox*^ mice (Figure 4*A–C*). Intriguingly, there were no tumors that invaded the submucosal area in any of these mice, in contrast to the scenario observed in *CPC;ApcPten* mice (Figures 3*D*, *E*, and 4*D*).

### Loss of Heterozygosity Occurred at *Apc* Locus, But Not at *Pten*, in *CPC;ApcPten* Mice

Although the phenotype of *CPC;ApcPten* mice in colonic tumor formation suggests a tumor-suppressor function of *Pten* in a haploinsufficient manner, there is another possibility that the remaining wild-type *Pten* allele was lost in colonic tumors, as previously reported for the *Apc* locus.^16^ We examined the genotypes of *Apc* and *Pten* in the adjacent normal jejunum, colon, and colonic neoplasia in *CPC;Apc* and *CPC;ApcPten* mice.

**Figure 5:**
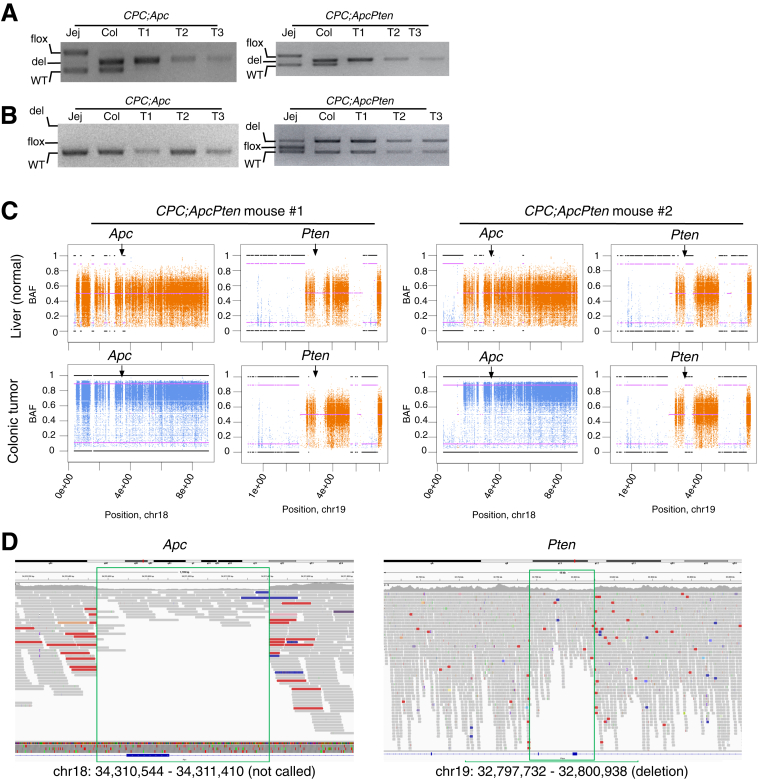
**WGS analysis reveals loss of heterozygosity at the *Apc* locus, but not the *Pten* locus in *CPC;ApcPten* mice.** (*A* and *B*) Genotyping of *Apc* (*A*) and *Pten* (*B*) in colonic tumors and adjacent normal jejunum and ileum in *CPC;Apc* and *CPC;ApcPten* mice. WT: wild-type allele. flox: flox allele without Cre recombination. del: inactivated flox allele via Cre. Jejunum (Jej), colon (Col), and tumor 1 (T1) were obtained from the same mouse, whereas tumor 2 (T2) and tumor 3 (T3) represent biological replicates from independent mice. (*C*) BAF calculated using Control-FREEC to call LOH in both normal tissue (liver) and colonic tumors in *CPC;ApcPten* mice. *Black and purple lines* show the expected and actual BAF values, respectively. *Orange and blue dots* represent the areas in which BAF = 0.5 and BAF = 0 or 1, respectively. In brief, LOH occurs in the area with *blue dots*. Shown are biological replicates. (*D*) Structural variants visualized using Integrative Genomics Viewer around the *Apc* and *Pten* loci in which floxed alleles are present. *Green boxes* indicate expected regions of deletion. The panel shows a representative case (#1).

Both wild-type and unrecombined floxed *Apc* alleles were observed in the jejunum, where *CDX2P* transcriptional activity is absent, in either *CPC;Apc* or *CPC;ApcPten* mice. In the adjacent normal colon, one floxed *Apc* allele was recombined (deleted), but there was a wild-type *Apc* allele. This wild-type allele was lost in colonic tumors in both *CPC;Apc* and *CPC;ApcPten* mice (Figure 5*A*), suggesting that LOH occurred. In contrast, the *Pten* wild-type allele remained in colonic tumors in *CPC;ApcPten* mice (Figure 5*B*), indicating that the loss of one copy of *Pten* was sufficient to facilitate a more aggressive phenotype in colonic tumorigenesis.

To further evaluate genomic alterations in *CPC;ApcPten* mice, analyses of copy number variants (CNVs) and LOH were performed using whole-genome sequencing (WGS) of *CPC;ApcPten*. We used the B-allele frequency (BAF) in Control-FREEC to investigate the loci at which LOH occurs. Briefly, the B allele indicates a different allele from the reference allele (namely, the A allele). If both alleles are intact, the BAF tends to show a value of 0.5, because of the heterozygosity of single nucleotide polymorphisms. However, the BAF shifts to 0 or 1 once LOH occurs. In this computational analysis using BAF, LOH occurred on chromosome 18, including *Apc* loci, specifically in colonic neoplasia (Figure 5*C*, *left*), whereas there were no significant changes in CNVs. This finding suggests copy-neutral LOH (cnLOH) in the mouse models, consistent with the occurrence of cnLOH in human colorectal adenocarcinomas.^38^ In contrast, there was no LOH in chromosome 19, including the *Pten* loci, in both normal liver and colonic tumors (Figure 5*C*, *right*), supporting haploinsufficiency of the *Pten* gene. We also confirmed a markedly decreased coverage of reads in reference mapping at the *Apc* locus, but there was only a slight decrease in coverage at the *Pten* locus (Figure 5*D*). In addition, there were no additional driver gene mutations in *CPC;Apc* or *CPC;ApcPten* mice identified using WGS.

### The Aggressive Phenotype in Colonic Tumor Formation in *CPC;ApcPten* Depends on Enhanced Mechanistic Target of Rapamycin Complex 1 Pathway by *Pten* Haploinsufficiency

**Figure 6:**
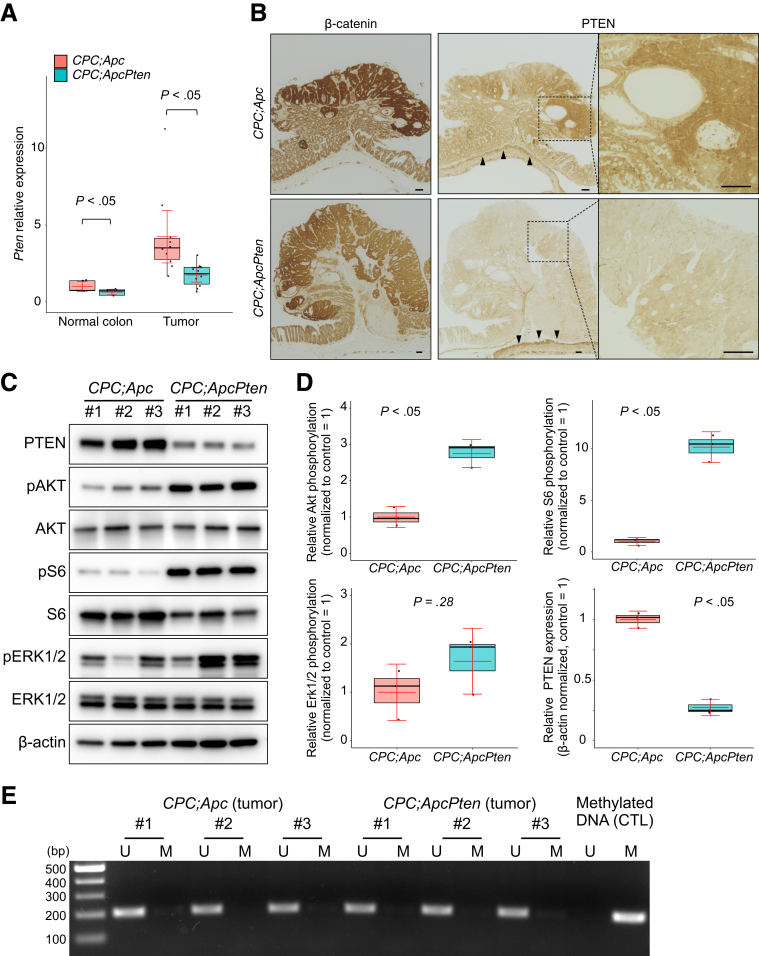
**Reduced PTEN expression and activation of downstream AKT–S6 signaling in *CPC;ApcPten* mice.** (*A*) qRT-PCR analysis of *Pten* in normal colon and colonic tumors in *CPC;Apc* and *CPC;ApcPten* mice. The expression level was normalized to to the level of β2 microglobulin as a housekeeping gene. n = 5 and 5 for normal colon, and n = 10 and 10 for colonic tumors in *CPC;Apc* and *CPC;ApcPten* mice, respectively. (*B*) Micrographs of immunohistochemical detection of β-catenin and PTEN in colonic tumors generated in *CPC;Apc* and *CPC;ApcPten* mice. Note that a muscular layer indicated by *arrowheads* was used as an internal control for PTEN immunoreactivity because *CDX2P-Cre* limited PTEN expression in colonic and intestinal epithelial cells. Although there was similar immunoreactivity in the muscular layer, there was decreased expression levels of PTEN in *CPC;ApcPten* mice compared with *CPC;Apc* mice. Scale bar: 100 μm. (*C*) Immunoblotting of PTEN, pAKT, AKT, pS6, S6, pERK, ERK, and a loading control β-actin in colonic tumors from *CPC;Apc* and *CPC;ApcPten* mice. Each lane represents an independent biological replicate. (*D*) Quantification of immunoblotting results. Data in (*A*) and (*D*) are presented as boxplots with individual data points; *red lines* indicate means with 95% CIs. (*E*) Methylation-specific PCR performed on DNA extracted from tumors of *CPC;Apc* and *CPC;ApcPten* (n = 3 biological replicates per genotype). Universal methylated mouse DNA Standard was used as the methylated DNA control (CTL). U and M denote amplification of unmethylated and methylated DNA, respectively.

Next, we investigated the effect of *Pten* haploinsufficiency on downstream signaling pathways. First, we confirmed decreased PTEN expression at the messenger RNA (mRNA) and protein levels in *CPC;ApcPten* mice compared with *CPC;Apc* mice (Figure 6*A–D*). In addition, the phosphorylation levels of ATK serine-threonine protein kinase (AKT) and S6 ribosomal proteins were increased in colonic tumors in *CPC;ApcPten* mice compared with *CPC;Apc* mice, whereas phosphorylation of extracellular signal-regulated kinases 1 and 2 (ERK1/2) varied among the samples, independent of the *Pten* genetic status (Figure 6*C* and *D*). We further examined whether epigenetic silencing contributes to the reduction of *Pten* expression in *CPC;ApcPten* mice. To this end, we performed a methylation-specific polymerase chain reaction (PCR) assay, which revealed that the unmethylated *Pten* promoter was predominant, indicating that promoter methylation is unlikely to account for decreased *Pten* expression (Figure 6*E*).

**Figure 7:**
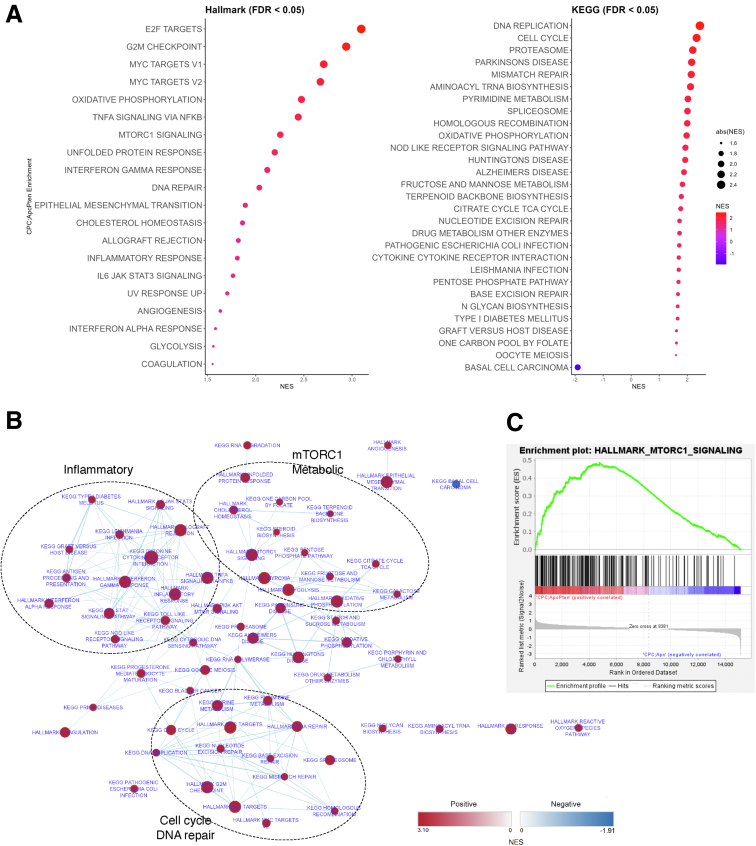
**Transcriptomic analysis reveals mTORC1 pathway activation in *CPC;ApcPten* tumors.** Summary of GSEA using RNA sequencing data comparing *CPC;ApcPten* mice with *CPC;Apc* mice (control). (*A*) Dot plots for Hallmark and KEGG pathways upregulated or downregulated in *CPC;ApcPten* mice. Cutoff; FDR <.05. (*B*) Pathway network visualized by The EnrichmentMap Cytoscape. Cutoff; FDR <.1. (*C*) GSEA enrichment plots for HALLMARK_MTORC1_SIGNALING (NES = 2.26 , FDR < .0001 , *P* < .0001). RNA sequencing analysis was performed using 3 biological replicates per group (*CPC;Apc* and *CPC;ApcPten*).

We performed RNA sequencing and subsequent gene set enrichment analysis (GSEA) to compare the colonic tumors in *CPC;ApcPten* mice with those in *CPC;Apc* mice. By setting the cutoff value as a false discovery rate (FDR) < .05, 20 and 28 signaling pathways were upregulated the in Hallmark and Kyoto Encyclopedia of Genes and Genomes (KEGG) databases, respectively, and one was downregulated in the KEGG database in *CPC;ApcPten* mice (Figure 7*A*). These signal pathways largely formed 3 clusters: mechanistic target of rapamycin complex 1 (mTORC1)-metabolic, inflammatory, and cell cycle–DNA repair-related (Figure 7*B*). Of these, we focused on the mTORC1 pathway (Figure 7*C*) because it is a canonical downstream signaling pathway of PTEN.

**Figure 8:**
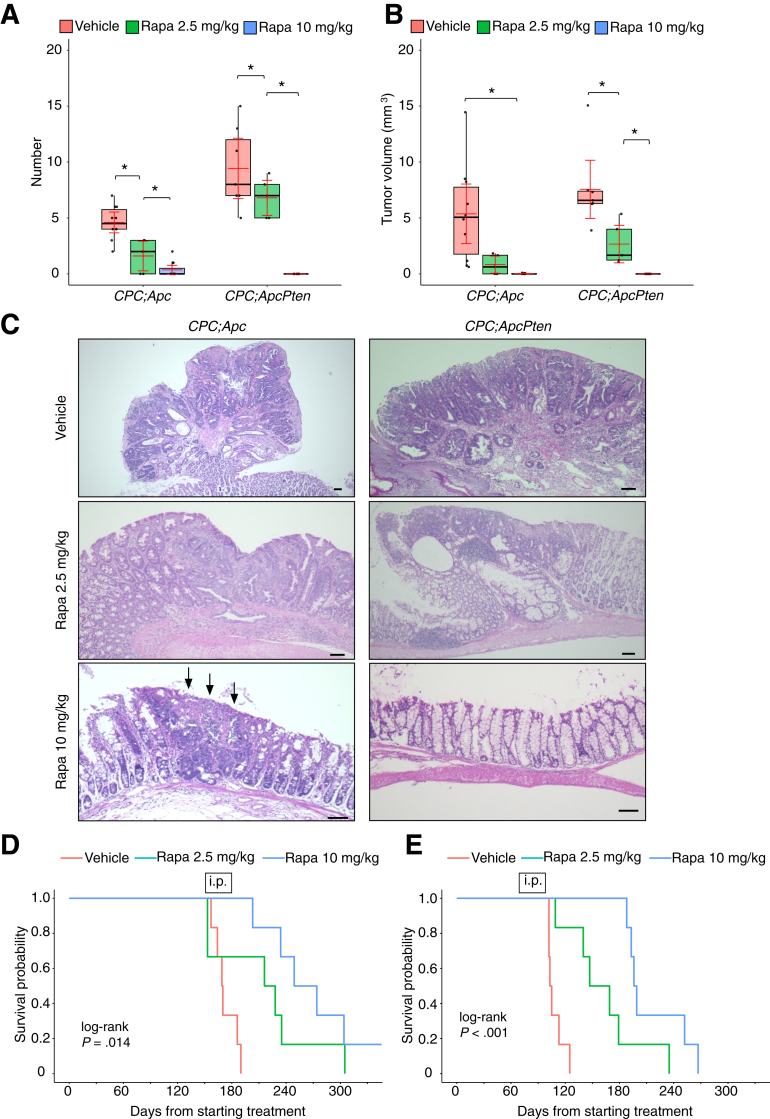
**Short-term rapamycin treatment suppresses invasive tumor growth and improves survival in *CPC;ApcPten* mice.** (*A–C*) A daily dose of 2.5 or 10 mg/kg of rapamycin or PBS as a vehicle control was intraperitoneally injected into *CPC;Apc* and *CPC;ApcPten* mice between 9 and 13 weeks of age. Colonic and intestinal tumor formation in *CPC;Apc* and *CPC;ApcPten* mice were compared at the end of treatment period. (*A* and *B*) Quantification of tumor number (*A*) and tumor volume (*B*). Data are presented as boxplots with individual data points, and *red lines* indicate means with 95% CIs. Asterisks indicate *P* < .05. n = 10, 5, and 11 for *CPC;Apc* mice and n = 7, 5, and 7 for *CPC;ApcPten* mice treated with 0, 2.5, and 10 mg/kg rapamycin, respectively. (*C*) Representative micrographs of H&E staining. Note that no tumors were observed in *CPC;ApcPten* mice treated with 10 mg of rapamycin, although small tumors were present in *CPC;Apc* mice, as indicated by the *arrows*. Scale bar: 100 μm. (*D* and *E*) Effects of rapamycin on survival in *CPC;Apc* (*D*) and *CPC;ApcPten* (*E*). *CPC;Apc* and *CPC;ApcPten* mice were treated with a daily dose of 2.5 or mg/kg rapamycin or vehicle from 150 days and 100 days of age, respectively, at which point, one-half of the mice died (see Figure 3*F*). Each group included 6 mice.

**Figure 9:**
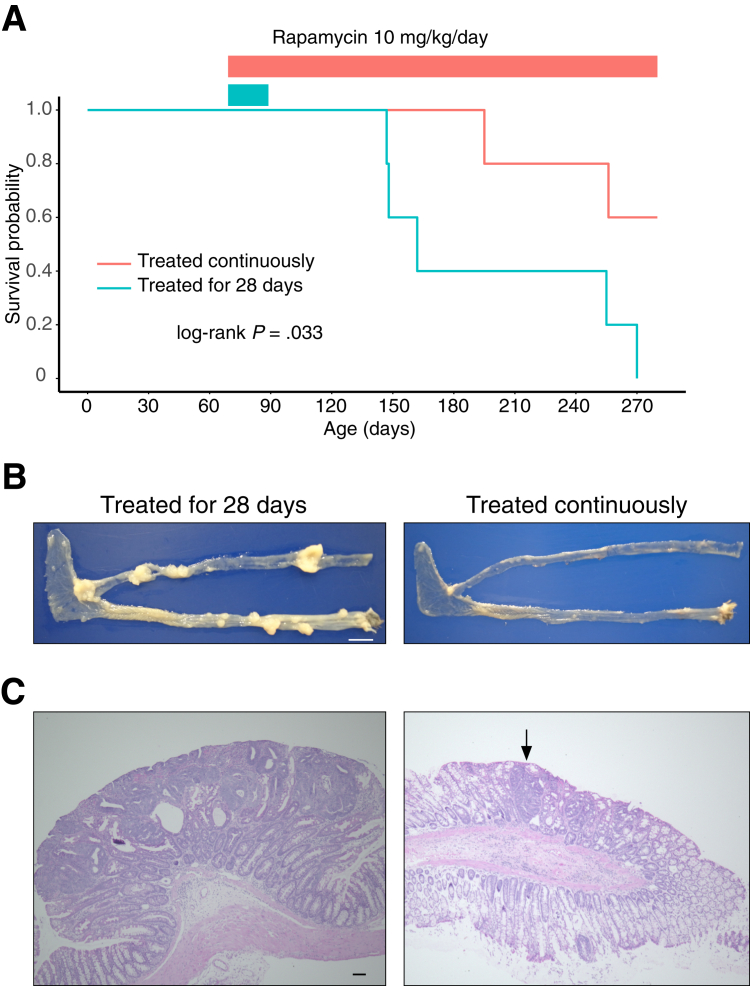
**Sustained rapamycin treatment prevents tumor recurrence in *CPC;ApcPten* mice.** Extended rapamycin administration sustainably inhibited tumorigenesis in *CPC;ApcPten* mice, although colonic adenocarcinoma developed again after stopping rapamycin treatment. (*A*) Comparison of survival between the 28 days of treatment and continuous treatment groups. (*B* and *C*) Representative gross pictures (*B*) and micrographs of H&E staining (*C*) of colonic tumors in both groups. Scale bar: 10 mm and 100 μm for (*B* and *C*), respectively. Each group consisted of 5 *CPC;ApcPten* mice.

Next, we treated the GEMMs with rapamycin to determine whether the tumors were dependent on the mTORC1 pathway. Consistent with previous reports showing that *Apc*-deficient intestinal tumors are sensitive to mTOR inhibition,^39^, ^40^, ^41^ tumor formation was decreased in *CPC;Apc* mice. Furthermore, rapamycin treatment significantly and dose-dependently reduced tumor number and burden, accompanied by prolonged lifespan, in *CPC;ApcPten* mice (Figure 8*A–D*). Notably, despite the higher baseline tumor incidence in *CPC;ApcPten* mice, rapamycin almost completely abolished tumor formation at 10 mg/kg, whereas a few tumors remained in *CPC;Apc* mice treated at the same dose. Furthermore, we continuously administered 10 mg of rapamycin to *CPC;ApcPten* mice over 6 months to test whether genetic alterations other than those in the mTOR pathway affected colonic tumorigenesis. However, tumor formation was profoundly suppressed, and only 1 microadenoma was formed in 1 of 3 *CPC;ApcPten* mice continuously treated with rapamycin (Figure 9*A–C*).

**Figure 10:**
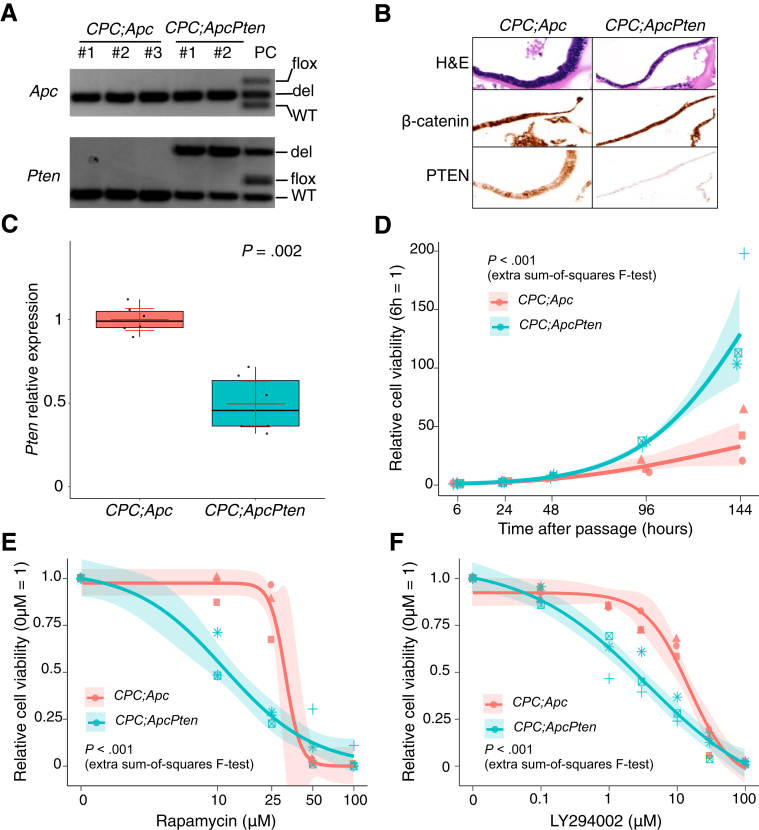
***CPC;ApcPten* tumor organoids show increased sensitivity to PI3K-mTORC1 pathway inhibitors compared with *CPC;Apc* tumor organoids.** (*A*) Genotyping of *Apc* and *Pten* in organoids derived from *CPC;Apc* (n = 3 biological replicates) and *CPC;ApcPten* (n = 2 biological replicates) tumors. (*B*) Micrographs of H&E and immunohistochemical staining for β-catenin and PTEN. PTEN expression is reduced in *CPC;ApcPten* organoids compared with *CPC;Apc*. (*C*) qRT-PCR analysis of *PTEN*. mRNA levels of *Pten* expression were compared between *CPC;Apc* and *CPC;ApcPten* tumor organoids (n = 6 biological replicates per genotype). (*D*) Growth curve analysis of *CPC;Apc* and *CPC;ApcPten* tumor organoids. Longitudinal growth curves were modeled using a 4-parameter log-logistic (LL.4) function and plotted as fitted trajectories with 95% CIs, based on 3 biological replicates per genotype. (*E* and *F*) Effect of PI3K-mTORC1 inhibition on colon tumor organoids derived from *CPC;Apc* and *CPC;ApcPten* mice. Organoids were treated with rapamycin (*E*) and LY294002 (*F*) for 5 days. Dose-response curves were fitted using a 4-parameter log-logistic model (LL.4) to estimate ED_50_ (equivalent to IC_50_) with 95% CIs.

Because rapamycin markedly suppressed tumor formation in both *CPC;Apc* and *CPC;ApcPten* mice, differences in mTORC1 pathway dependency were difficult to distinguish based solely on in vivo responses. To more quantitatively assess pathway dependence, we established colonic tumor organoids from each genotype as an ex vivo model. We first confirmed that *Pten* expression remained haploinsufficient in *CPC;ApcPten* organoids (Figure 10*A–C*). We also observed *CPC;ApcPten* tumor organoid proliferates more rapidly than *CPC;Apc* organoids (Figure 10*D*), supporting the greater tumor number and burden observed in vivo. We then performed dose-response analysis for rapamycin, which demonstrated a clear left-shift in sensitivity: effective dose achieving 50% inhibition (ED_50_) = 11.9 ± 2.3 μM for *CPC;ApcPten* organoids vs 31.5 ± 4.0 μM for *CPC;Apc* organoids (*P* < .001, F-test) (Figure 10*E*). A comparable pattern was also observed with the PI3K inhibitor LY294002, in which the estimated ED_50_ was approximately 3.56 ± 1.9 μM for *CPC;ApcPten* organoids and 14.8 ± 2.5 μM for *CPC;Apc* organoids (*P* < .001, F-test) (Figure 10*F*). Taken together, these ex vivo findings complement the in vivo data by demonstrating quantitatively increased reliance on PI3K-mTOR signaling when *Pten* expression is reduced.

### Phylogenetic Analyses of Tumor Evolution in Human Colorectal Neoplasia by Targeted Biopsy Under Colonoscopy Reveals the Association of PI3K Pathway Abnormality with Submucosal Invasion

**Figure 11:**
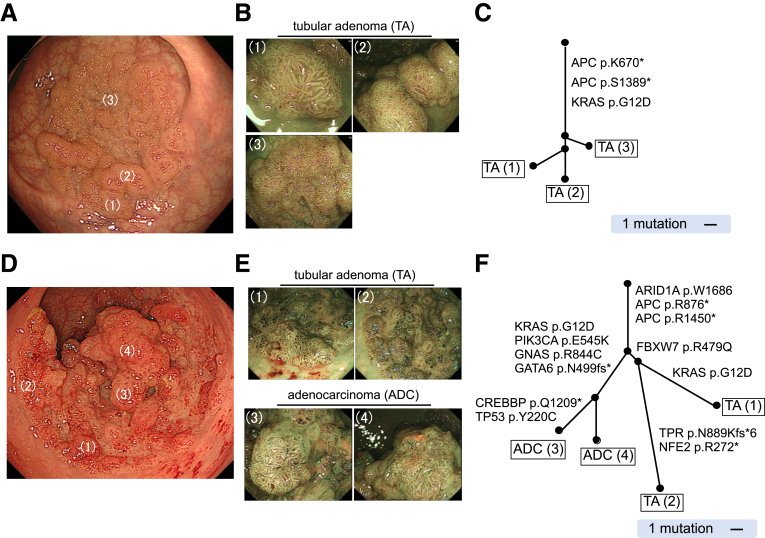
**Clonal evolution of human colorectal neoplasia revealed by phylogenetic analysis of multiple targeted biopsies.** (*A–F*) Two representative cases of human colorectal neoplasia. (*A* and *D*) Regular colonoscopic images. (*B* and *E*) Magnified NBI corresponding to the numbers indicated in Figure (*A* and *D*), respectively. Targeted biopsies were performed in each site. Histological prediction was based on the JNET classification. Areas (1)–(3) in (*B*) were classified as Type 2A because of regular vessel and surface patterns. In (*E*), areas (1) and (2) were also classified as Type 2A, whereas areas (3) and (4) were diagnosed as Type 2B due to irregular vessel and surface patterns. (*C* and *F*) Phylogenetic trees of tumor evolution.

**Figure 12:**
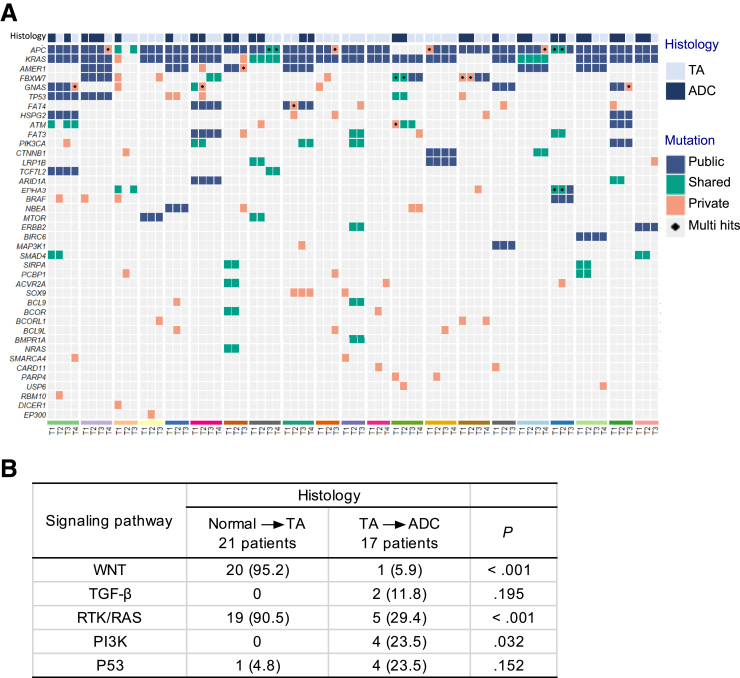
**Landscape of genetic alterations associated with the adenoma-carcinoma sequence in human colorectal neoplasia.** (*A*) The landscape of genetic alteration in 21 cases of human colorectal neoplasia. Several biopsies were performed at different sites in the same tumors for DNA sequencing. Public: genetic alterations existing in all regions of the tumor, shared: genetic alterations existing in part of all regions, private: genetic alterations existing in a single region. (*B*) Association of genetic alterations with the transition from normal epithelium to adenoma and from adenoma to adenocarcinoma.

Our data established a haploinsufficient effect of the *Pten* gene on colonic tumor formation in mice. To implicate genetic alterations in the PI3K-AKT-mTOR signaling pathway in human colorectal carcinogenesis, we examined the clonal evolution of human colorectal neoplasia using multiple biopsies under colonoscopy, and used magnified NBI to predict histology based on surface patterns (Figure 11*A*, *B*, *D* and *E*). We performed next-generation DNA sequencing of these biopsy specimens, followed by phylogenetic analysis of tumor evolution (Figures 11*C*, *F*, and 12*A*). The results revealed that WNT/β-catenin and receptor tyrosine kinase (RTK)/RAS abnormalities mostly occurred in the transition from normal epithelium to tubular adenoma. In contrast, PI3K pathway abnormalities were predominantly observed in the transition from tubular adenoma to submucosal-invasive adenocarcinoma, suggesting an association between PI3K pathway abnormalities and the invasive phenotype of human colorectal neoplasia (Figure 12*A* and *B*).

**Figure 13:**
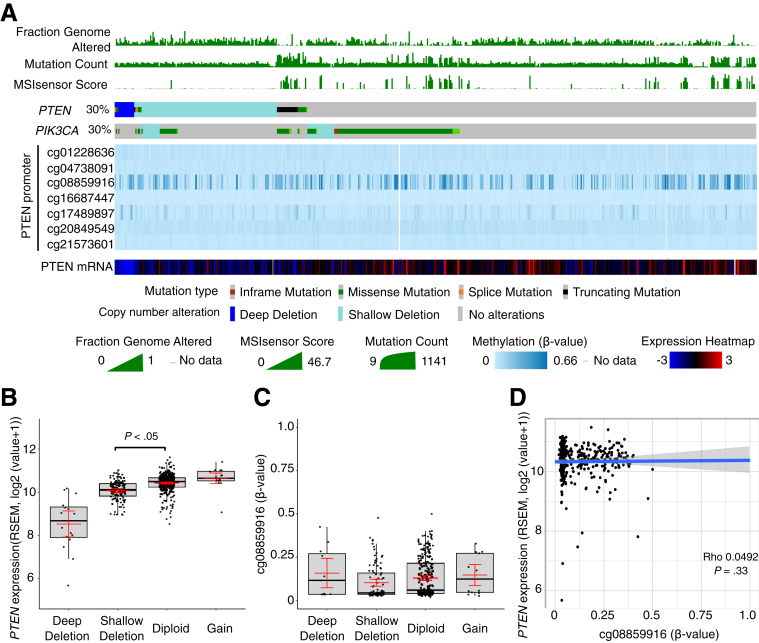
**Association among copy number, promoter methylation, and mRNA expression of PTEN in human colorectal cancer.** (*A*) Oncoprint for *PTEN* and *PIK3CA* in the TCGA-COADREAD dataset. (*B*) Correlation between mRNA level and the copy number status of *PTEN*. (*C*) Correlation between promoter methylation level at cg08859916 and the copy number status of PTEN. (*D*) Correlation between the promoter methylation level at cg08859916 and the mRNA levels of *PTEN*. Data are shown as boxplots with individual data points and *red lines* indicating means with 95% CIs in (*B*) and (*C*), and as scatter plots with linear regression lines and 95% confidence bands in (*D*).

We further investigated genomic and epigenomic alterations in the *PTEN* locus using The Cancer Genome Atlas- Colon Adenocarcinoma and Rectal Adenocarcinoma (TCGA-COADREAD) data. As shown in Figure 14*A*, the landscape of mutations, copy-number alterations, promoter methylation, and mRNA expression was visualized across CRCs. *PTEN* mRNA expression significantly decreased when the copy number changed from 2 to 1 (Figure 13*B*), indicating a dosage-dependent effect of heterozygous loss. However, *PTEN* promoter methylation did not correlate with either copy-number status or mRNA expression levels (Figure 13*C* and *D*). These results suggest that reduced *PTEN* expression in human CRC is mainly attributable to copy-number loss rather than promoter hypermethylation, consistent with the phenotype observed in our *Pten*-haploinsufficient mouse model.

## Discussion

Since multistep carcinogenesis in the colon was first proposed,^1^ many cancer-associated genes have been identified.^3^ GEMMs have been instrumental in dissecting genotype-phenotype correlations in colorectal tumorigenesis.^12^^,^^13^ In recent years, increasingly sophisticated GEMMs have been developed to model stepwise tumor progression and to examine how specific combinations of driver mutations influence invasion and malignancy. In this study, we comprehensively studied various combinations of driver genes for colonic tumor formation in GEMMs using colonic epithelial cell-specific *CDX2P-Cre* mice. In this series of GEMM studies, we found that *CPC;ApcPten* mice developed invasive adenocarcinomas in mouse colon very efficiently.

Consistent with the pivotal role of the PI3K-mTOR pathway, several previous studies have demonstrated that activation of this signaling axis promotes invasion or malignant progression in intestinal tumors. Specifically, *Adeno-Cre*-mediated activation of *Pik3ca* induces invasive lesions in the context of concomitant *Apc* loss,^22^ whereas *Pik3ca* activation in *Villin-Cre*- or *Villin-CreERT*-based intestinal models has been associated with invasive and metastatic phenotypes.^42^ In addition, AOM-assisted Akt activation enhances tumor progression,^24^ and concurrent *Kras* mutation with *Pten* loss leads to serrated adenoma formation accompanied by strong mTOR activation.^18^ Building upon these findings, our colon-restricted *CPC;ApcPten* model demonstrates that even partial, heterozygous loss of *Pten* is sufficient to drive invasive adenocarcinomas in the colon under purely genetic and tissue-restricted conditions.

Reinforcing these experimental findings, mutations in the PI3K pathway, particularly in *PIK3CA* and *PTEN*, have been implicated in the late adenoma-to-adenocarcinoma transition in human colorectal tumorigenesis.^2^ Although this concept was originally based on histological analyses of premalignant polyps, the causal evidence has remained limited. *PTEN* acts as a tumor suppressor by antagonizing PI3K signaling and maintaining genomic stability.^43^ However, accumulating evidence suggests that even partial reduction of PTEN function can promote malignant progression. For example, *Apc*^*min/+*^*;Pten*^*+/–*^ mice developed invasive adenocarcinomas in the small intestine,^36^ supporting the concept of PTEN haploinsufficiency. Nevertheless, whether *Pten* haploinsufficiency alone can drive invasive transformation in the colon remained untested.

To address this question, in our colon epithelium-specific *CPC;ApcPten* model, we confirmed that the remaining wild-type *Pten* allele was intact, and transcriptomic analyses revealed enhanced PI3K-mTOR pathway activity in a haploinsufficient manner. This provides the first direct in vivo evidence that partial *Pten* loss is sufficient to induce invasive adenocarcinoma specifically in the colon. Of note, osteopontin was not differentially expressed in our RNA-sequencing data, likely reflecting differences between global and epithelial-specific knockouts. Given that osteopontin-expressing tumor-associated macrophages play critical roles in the CRC microenvironment,^44^^,^^45^ our model—where stromal cells retain wild-type *Pten*—offers a valuable system to study tumor-immune interactions during PI3K-mTOR-driven invasion.

We further demonstrated that loss of one copy of the *Pten* gene was sufficient to promote tumor formation and invasion through mTORC1 activation, as evidenced by the strong therapeutic response to rapamycin. As expected, tumors showed near-complete regression upon rapamycin administration in *CPC;ApcPten* mice (Figure 8*A–E*). However, we also observed a milder inhibitory effect in *CPC;Apc* mice, consistent with previous reports describing mTOR inhibition in *Apc*-deficient models.^39^^,^^40^ Moreover, organoid-based assays revealed that *CPC;ApcPten*-derived tumor organoids were more susceptible to rapamycin than those from *CPC;Apc* mice, indicating that partial *Pten* loss confers enhanced PI3K-mTORC1 dependency (Figure 10*D* and *E*).

We have reported that LOH due to chromosomal instability occurred in an intact *Apc* allele in *CPC;Apc* mice, resulting in colonic tumor formation.^16^ However, the mode of LOH has not been elucidated well. Although classical LOH indicates the deletion of a large area of the chromosome, cnLOH is a variation of LOH accompanied by a normal copy number of chromosomes.^46^^,^^47^ Moreover, cnLOH is associated with carcinogenesis in CRCs.^38^^,^^46^ In a study of 5 patients with familial colonic polyposis, Li et al reported that cnLOH of *Apc* occurred in 3 of the patients,^48^ suggesting that cnLOH is a common genomic event during human colorectal carcinogenesis. Consistent with these findings, WGS revealed that cnLOH also occurs in colonic neoplasia in *CPC;ApcPten* mice. In contrast, the wild-type allele of the *Pten* gene was still intact in tumors of *CPC;ApcPten* mice, suggesting that the aggressive phenotype we observed was derived from the loss of only one copy of the *Pten* gene.

Furthermore, genomic analyses by Li et al demonstrated that *PIK3CA* mutations were associated with the transition from adenoma to adenocarcinoma.^48^ In our cohort of human colonic neoplasia, genetic alterations of *PI3K* were also frequently observed when adenomas turned into adenocarcinomas. However, most of these mutations were PIK3CA mutations. Heterozygous loss of *PTEN* has not been evaluated using cancer genome profiling. Although homozygous and heterozygous losses of *PTEN* are seen in 3% and 22% of cases, respectively, in the TCGA-COADREAD dataset, the impact of heterozygous loss of *PTEN* on human CRC progression and prognosis remains unclear. The mRNA levels of *PTEN* were lower in the heterozygous loss group than in the diploid group. Because there is no apparent promoter methylation, these expression changes seem to be a result of the copy number alteration itself (Figure 13). In future studies, clinical whole-exome sequencing or WGS to study copy number alteration will be useful to determine the function of dose-dependent tumor-suppressor genes, such as *PTEN*.

In summary, we showed that the loss of one copy of *Pten* with inactivation of *Apc* in colonic epithelial cells resulted in the formation of a tumor invading the submucosa of the mouse colon, and this was mediated by the upregulation of mTORC1 signaling through *PTEN* haploinsufficiency. We also present the evolutionary trajectories from tubular adenomas to adenocarcinomas using multiple targeted biopsies under colonoscopy in cases of adenocarcinoma of the human colon and rectum. *APC* and *KRAS* were frequently mutated during tubular adenoma formation, whereas mutations in the PI3K pathway were associated with the transformation from adenoma to adenocarcinoma in human colonic tumorigenesis. Based on our mouse and clinical studies, we conclude that alterations in the PI3K-mTOR signaling pathway increase the rate of adenocarcinoma occurrence.

## Methods

### Mice

All animal procedures were approved by the Committee of Animal Experimentation, Hiroshima University (A16-40), and were conducted in accordance with the Guidelines of the Committee of Animal Experimentation and the Committee of Research Facilities for Laboratory Animal Science, Natural Science Center for Basic Research and Development (N-BARD), Hiroshima University. *CDX2P 9.5-NLS-Cre*, *CDX2P-G22-Cre*, and *Apc*^*flox*^ mice were generated as described previously.^16^^,^^28^ *Pten*^*flox*^ mice were obtained from the Jackson Laboratory. The primers used for PCR genotyping are listed in Table 1. All genetically engineered mice used in this study were maintained on a C57BL/6J background under specific pathogen-free conditions at Hiroshima University. Mice were housed in individually ventilated cages in a temperature-controlled room (22°C ± 2°C) with a 12-hour light/dark cycle and provided free access to standard chow and water. Experimental and control littermates were randomly assigned to treatment groups. Investigators were not blinded to allocation during experiments, but data quantification was performed in a blinded manner when applicable. Sample sizes were determined based on our previous experience with similar models to achieve consistent detection of significant differences in tumor number and burden while minimizing animal use. Both male and female mice were used unless otherwise specified. No sex-specific differences in tumor incidence, histopathology, or response to rapamycin were observed, and therefore data from both sexes were combined for statistical analysis. Rapamycin (Sigma-Aldrich) was dissolved in phosphate-buffered saline (PBS) and administered via intraperitoneal injection. Mice were euthanized using carbon dioxide gas and subsequent cervical dislocation for tumor harvesting, or when they met the humane endpoints for euthanasia, such as 20% of body weight loss from baseline, severe rectal prolapse, or severe bleeding due to tumor development.

**Table 1:** PCR Primers

Primer	Sequence (5′–3′)
Genotyping primers	
*Apc*_flox P3	GTTCTGTATCATGGAAAGATAGGTGGTC
*Apc*_flox P4	CACTCAAAACGCTTTTGAGGGTTGATTC
*Apc*_flox P5	GAGTACGGGGTCTCTGTCTCAGTGAA
*Pten*_a	GGCCTAGGACTCACTAGATAGC
*Pten*_b	GTGAAAGTGCCCCAACATAAGG
*Pten*_c	CTCCCACCAATGAACAAACAGT
qRT-PCR primers	
*Pten*_Forward	AAGTGGAGACAGGCTGATGTG
*Pten*_Reverse	TGTTGCCACAAGTGCAAAGG
Methylation-specific PCR primers	
*Pten*_Unmethylated_Forward	GGTTAGTTTTATGGGTTATAAGTGA
*Pten*_Unmethylated_Reverse	TCAAAACAAAAAATAACATCCACAA
*Pten*_Methylated_Forward	GGTTAGTTTTATGGGTTATAAGCGA
*Pten*_Methylated_Reverse	GAAACAAAAAATAACATCCACGAA

PCR, polymerase chain reaction; qRT-PCR, quantitative reverse transcriptional polymerase chain reaction.

### Organoids

Freshly harvested tumors were minced and incubated in 1 mM ethylenediaminetetraacetic acid (EDTA)/ethylene glycol-bis(2-aminoethylether)-N,N,N′,N′-tetraacetic acid (EGTA) /0.1 mM dithiothreitol in PBS at 4°C for 30 minutes. Subsequently, the tumors were digested with 2 mg/mL collagenase II (Worthington Corp) at 37°C for 45 minutes and then passed through a 70-μm filter. Isolated tumor cells were embedded in Matrigel (Corning Life Science) and cultured in basal tumor growth media containing advanced Dulbecco’s Modified Eagle Medium (DMEM)/F-12 with GlutaMAX, N2, and B27 supplements (Thermo Fisher Scientific), 50 ng/mL recombinant mouse epidermal growth factor (EGF; R&D Systems), and 1% antibiotic-antimycotic (Corning Life Science). Organoid proliferation was assessed as a time-course luminescence-based assay using CellTiter-Glo 3D (Promega). To evaluate PI3K-mTOR pathway dependence, dose-response analyses were performed with rapamycin (Sigma-Aldrich) and LY294002 (Selleck Chemicals) for 5 days.

### Endoscopic Evaluation and Histopathological Prediction Using NBI

Histopathological prediction using magnifying endoscopy with NBI was performed according to the Japan NBI Expert Team (JNET) classification.^49^ The magnifying endoscopic diagnosis was independently conducted by 2 board-certified endoscopists of the Japan Gastroenterological Endoscopy Society, and the final diagnosis was determined by consensus between them.

### Tissue Preparation for Staining, Histological Analysis, and Immunohistochemistry

All mouse specimens and organoids were fixed in 4% paraformaldehyde in PBS and were processed into paraffin blocks. Five-micrometer sections were then prepared and hematoxylin and eosin (H&E) staining was performed for histological analysis. For immunohistochemistry, heat-induced epitope retrieval methods were performed using citrate buffer (pH 6) and peroxidase activity was blocked with 3% hydrogen peroxide in methanol. Immunohistochemistry for β-catenin was performed using an M.O.M. Immunodetection Kit (Vector Laboratories) as previously described.^50^ For immunohistochemical analysis of PTEN, the sections were blocked with 5% normal donkey serum and 3% bovine serum albumin (BSA) at 22°C for 1 hour, after which, the sections were incubated with primary and secondary antibodies. All antibodies used for immunochemistry are listed in Table 2.

**Table 2:** Antibodies for Immunofluorescence and Immunoblotting

Antigen	Clone	Supplier (catalog number)	Dilution	Host
Immunofluorescence				
β-Catenin	14/β-Catenin	BD Transduction Laboratories (#610153)	1:200	Mouse
PTEN	Polyclonal	Cell Signaling (#9552)	1:100	Rabbit
M.O.M. anti-mouse IgG Reagent, Biotinylated	Polyclonal	Vector Laboratories (MKB-2225)	1:250	Horse
Rabbit IgG (H+L), HRP	Polyclonal	ThermoFisher Sceintific (A16023)	1:250	Donkey
Immunoblotting				
PTEN	Polyclonal	Cell Signaling (#9552)	1:1,000	Rabbit
AKT	Polyclonal	Cell Signaling (#9272)	1:1,000	Rabbit
Phospho-AKT (Ser473)	D9E	Cell Signaling (#4060)	1:1,000	Rabbit
S6 Ribosomal Protein	5G10	Cell Signaling (#2217)	1:1,000	Rabbit
Phospho-S6 Ribosomal Protein (Ser235/236)	D57.2.2E	Cell Signaling (#4858)	1:1,000	Rabbit
p42/44 MAPK (Erk1/2)	137F5	Cell Signaling (#4695)	1:1,000	Rabbit
Phospho-p42/44 MAPK (Erk1/2) (Thy202/Tyr204)	D13.14.4E	Cell Signaling (#4370)	1:1,000	Rabbit
Rabbit IgG (H+L), HRP	Polyclonal	ThermoFisher Sceintific (A16023)	1:5,000	Donkey
Mouse IgG (H+L), HRP	Polyclonal	Proteintech (SA00001-8)	1:5,000	Donkey

### Immunoblotting

Mouse specimens were lysed in radioimmunoprecipitation assay buffer with protease and phosphatase inhibitors using sonication at 4°C. The lysates were boiled in 4× Laemlli sample buffer (Bio-Rad Laboratories) and used for sodium dodecyl sulfate-polyacrylamide gel electrophoresis (SDS-PAGE) and immunoblotting. Blocking was performed in 5% milk with 0.1% Tween-20 in Tris-buffered saline for 1 hour at 22°C. Membranes were incubated with primary antibody at 4°C overnight and then with a horseradish-peroxidase (HRP)-conjugated secondary antibody for 1 hour at 22°C. Chemiluminescence was detected using an Amersham ImageQuant 800 (Cytiva). Band intensities in immunoblots were quantified using ImageJ software (National Institutes of Health). The antibodies used in this study are listed in Table 2.

### Quantitative Reverse Transcriptional PCR

RNA was extracted from mouse specimens and organoids using an RNeasy Mini Kit (Qiagen). Complementary DNA (cDNA) was generated from the extracted RNA using an iScript cDNA Synthesis Kit (Bio-Rad Laboratories), and real-time PCR for *Pten* was performed using SsoAdvanced Universal SYBER Green Supermix and a Bio-Rad CFX96 instrument (Bio-Rad Laboratories). The primers used for quantitative reverse transcriptional polymerase chain reaction (qRT-PCR) are listed in Table 1.

### WGS

DNA was extracted from the frozen samples using NucleoBond HMW DNA (TaKaRa Bio). Sequence analysis was performed using the DNBSEQ platform at a depth of 100×. Pre-processing of the sequence reads was performed using fastp v0.23.2, followed by alignment to the mm10 reference genome using BWA-MEM v0.7.17. Variant calling was conducted based on the best practices of the Gene Analysis Toolkit. To remove false positives, a cutoff of depth ≥30 and variant allele frequency (VAF) >0.06 were applied. Variant annotation was performed using vcf2maf v1.6.21 (Cyriac Kandoth. mskcc/vcf2maf: vcf2maf v1.6.19. (2020). https://doi.org/10.5281/zenodo.593251). IGV v2.10.0 was employed for alignment visualization, and Manta v1.6.0 was used to call the structural variants. Control-FREEC v11.6 was utilized for CNV and LOH analyses. Qualimap v2.2.2a and MultiQC v1.12 were employed for quality checks.

### RNA Sequencing

RNA was extracted from frozen samples using the RNeasy Mini Kit (Qiagen). Sequence analysis was performed using the DNBSEQ platform at a depth of 100×. Preprocessing of the sequence reads was performed using fastp v0.23.2, followed by alignment to the mm10 reference genome and read counting using RSEM v1.3.3. STAR was selected as the aligner. The read counts were normalized using the DESeq2 R package and subjected to variance-stabilizing transformation for input into GSEA. MultiQC v1.12 was employed for quality checks. Enrichment analysis was conducted using GSEA software v4.1.0 (https://www.gsea-msigdb.org/). The gene sets used for analysis were from the Hallmark and C2 KEGG pathway databases. The results were plotted using the ggplot2 R package. Furthermore, a network representing overlaps among enriched pathways was constructed using Cytoscape v3.8.2 and the EnrichmentMap v3.3.6 plugin.

### DNA Methylation Analysis

Bisulfite conversion was performed on DNA extracted from mouse tumors using EZ DNA Methylation-Lightning Kit (Zymo Research). Subsequently, methylation-specific PCR was conducted with the primers listed in Table 1. Universal methylated mouse DNA Standard (Zymo Research) was used as a positive control.

### Statistical Analyses

All statistical analyses were performed using R (version 4.2.2; R Foundation for Statistical Computing) and JMP Pro 18 (SAS Institute). For continuous variables, nonparametric tests were used as appropriate. Box plots display individual data points and superimpose means and standard deviations (SDs) in accordance with journal guidelines. Categorical variables were analyzed using Fisher’s exact test. Survival curves were generated by the Kaplan-Meier method, and statistical differences were evaluated using the log-rank test. A *P* value of < .05 was considered statistically significant. For analyses of tumor number and volume, data were aggregated per mouse, and the mean tumor volume per animal was treated as one independent data point to account for intra-mouse clustering. For organoid assays, longitudinal growth data were fitted using a 4-parameter log-logistic model (LL.4; drc package in R), and fitted trajectories with 95% confidence intervals (CIs) were visualized. For analyses of dose-response relationships, relative cell viability was also modeled using a 4-parameter log-logistic function (LL.4) to estimate the ED_50_ (equivalent to IC_50_) with 95% CIs. Differences between genotypes were assessed using an extra sum-of-squares F-test, in which a shared-parameter model was compared with a genotype-specific model to determine whether the latter provided a significantly improved fit. Curves were plotted with confidence bands.

## Declaration of AI and AI-Assisted Technologies in the Writing Process

During the preparation of this work, the authors used OpenAI’s ChatGPT in order to assist with language editing and improving clarity of expression. After using this tool, the authors reviewed and edited the content as needed and take full responsibility for the content of the publication.

## Supplementary Material

Supplementary Material

Extended PDF
