# Supplementary figures and images for: Colon-Restricted Phosphatase and Tensin Homolog Deleted From Chromosome 10 Haploinsufficiency Models Phosphoinositide 3-Kinase Pathway-Driven Invasion in Colorectal Cancer
Source: Cell Mol Gastroenterol Hepatol. 2026 Mar 25;20(9):101773. doi: 10.1016/j.jcmgh.2026.101773 (PMC13331976; doi:10.1016/j.jcmgh.2026.101773)

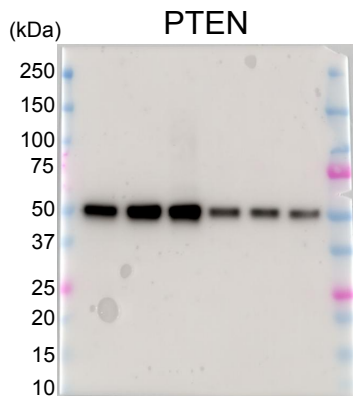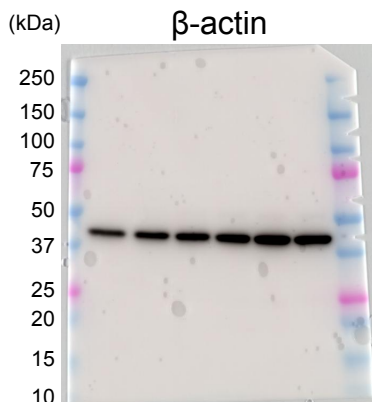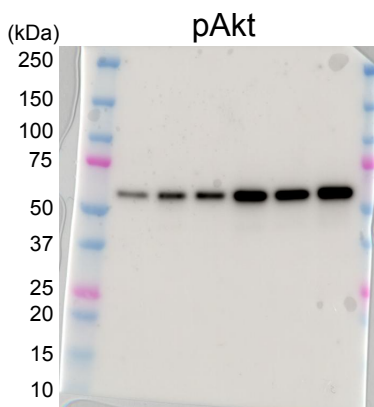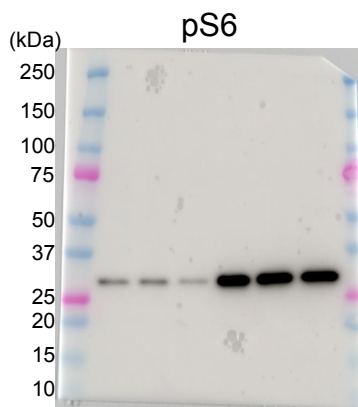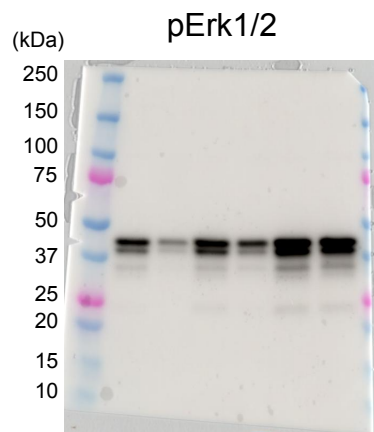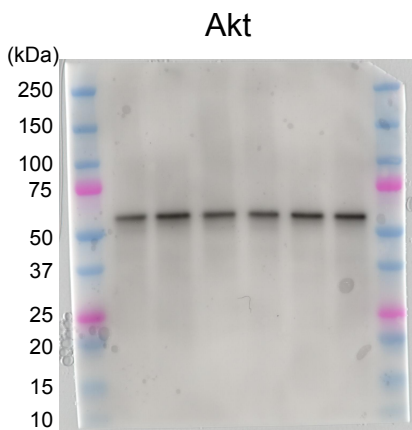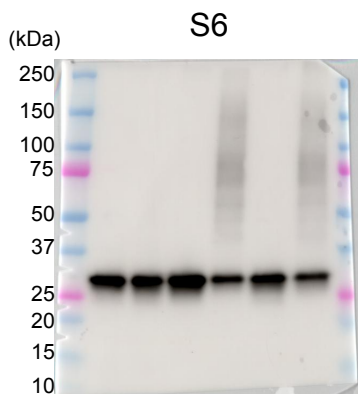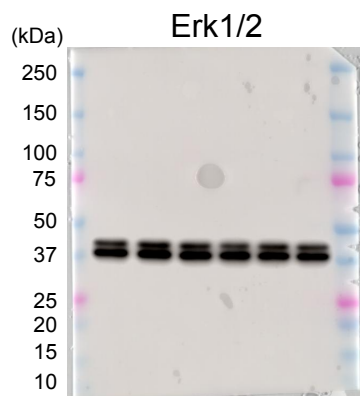

Supplement: Supplementary Material [file mmc1.pdf]
